# Dermatological care of gender‐diverse patients in Australia

**DOI:** 10.1111/ajd.14360

**Published:** 2024-08-08

**Authors:** Roy Kingsley Wong, Jenny Harrington, Annabel Irene Stevenson

**Affiliations:** ^1^ Faculty of Health and Medical Sciences University of Adelaide Adelaide South Australia Australia; ^2^ Division of Endocrinology Women's and Children's Hospital Adelaide South Australia Australia; ^3^ Department of Dermatology Royal Adelaide Hospital Adelaide South Australia Australia; ^4^ Department of Dermatology Queen Elizabeth Hospital Adelaide South Australia Australia

**Keywords:** Australia, cosmetic techniques, dermatology, sexual and gender minorities, skin diseases

## Abstract

In recent years, there has been an increasing recognition of the unique healthcare needs of gender‐diverse patients in Australia. With the continuous growth of referrals to gender health services, there is an increased demand for specialised dermatological care. There is still a significant knowledge gap and a lack of guidelines specifically tailored to this patient group. In this article, we will provide a brief overview of the journey of Transgender and Gender Diverse (TGD) individuals as they embark on psychological and pharmacologic treatment for gender dysphoria in Australia. We endeavour to contribute to the existing body of knowledge by examining the evidence surrounding the treatment of skin, hair and nail issues for TGD patients. This article will outline how dermatologists can assist in the care of the gender‐diverse patient. Although puberty blockade (stage 1 treatments) has minimal dermatological impact, gender‐affirming pharmacotherapy (stage 2 treatments) can lead to many dermatological issues including acne, patterned hair loss (PHL) and dermatitis. The dermatologist may also play a role in stage 3 treatments which include surgical or procedural interventions for gender affirmation.

## INTRODUCTION TO GENDER DIVERSITY MEDICINE IN AUSTRALIA

Gender diversity is an important part of Australia's history. For example, some First Nations communities have long‐adopted diverse views of gender that expand beyond westernised, binary representations of gender. In Australian First Nations communities, the terms Brotherboy and Sistergirl refer to individuals who have male or female spirit, and this spirit differs from their gender assigned at birth.

**FIGURE 1:**
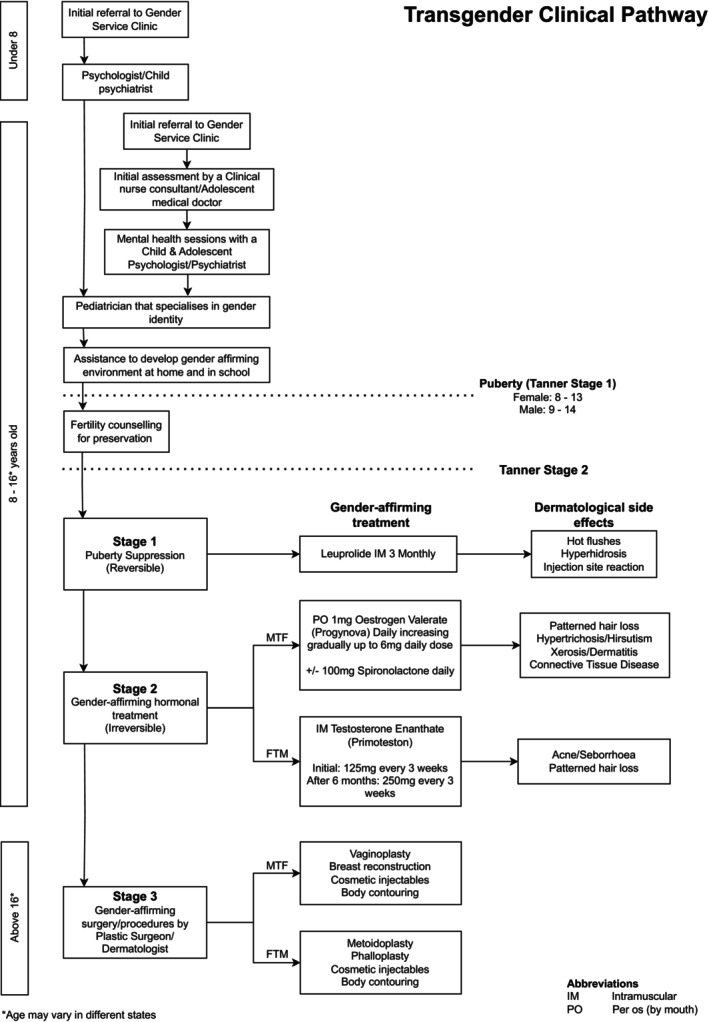
Gender‐diverse clinical pathway. Adapted from Telfer and The Royal Children's Hospital Melbourne Gender Service.^1^, ^7^

Gender diversity is sometimes accompanied by gender dysphoria, a form of distress that arises when there are incongruencies between a person's gender identity and their gender assigned at birth.^1^ At this time, studies have estimated that 1.2% of adolescents identify as TGD.^1^ Gender services now exist in all states and territories of Australia, highlighting an increasing demand for specialised dermatology care and a need for dermatologists to be aware of the specific considerations of treating this patient population.^2^ The flow chart (Figure 1) outlines the typical multidisciplinary pathway for a TGD individual experiencing gender dysphoria. The recommended interventions, including pharmacotherapies and procedures, at different stages of the gender transition are displayed alongside potential dermatological complications. This highlights the valuable role dermatologists can play within the TGD treatment. Important considerations include fertility preservation especially for TGD individuals transitioning from male to female (MTF), as oestrogen may render them infertile.

**Stage 1 treatment**, known as ‘puberty blockers’ (i.e. Leuprolide or Triptorelin, a gonadotropin‐releasing hormone agonist) can provide an extension to the temporal window for gender clarification, preventing the development of irreversible gender characteristics, and to alleviate gender dysphoria that can be exacerbated by the hormonal changes that occur during puberty.^3^, ^4^ Linear growth and weight gain continues during administration.

Once TGD individuals reach the appropriate age to provide informed consent (usually 15–16 years of age depending on state/territory and patient maturity), **stage 2 treatment** may be initiated. This is gender‐affirming partially irreversible medical therapy (oestrogen ± androgen blocker or testosterone). **Stage 3 treatment** involved surgical or procedural interventions such as vaginoplasty, phalloplasty, body contouring or cosmetic injectables.^5^ In Australia, irreversible surgical therapies are reserved for adult patients, with many patients travelling to Thailand for procedures, where there is regional expertise.^6^

Although gender‐affirming therapy aims to improve comfort and well‐being in TGD individuals, adverse effects from medical treatments and improper or illegal surgical interventions can negatively impact the skin, hair and nails.

## CONSIDERATIONS FOR DERMATOLOGICAL CARE IN GENDER‐DIVERSE PATIENTS

### Stage 1 treatment

Leuprolide, a GnRH agonist, initially stimulates the release of Luteinising Hormone (LH) and Follicle‐Stimulating Hormone (FSH) from the pituitary gland, leading to a transient increase in oestrogen and testosterone levels.^8^ With continuous use, it suppresses these hormones. This hormonal fluctuation affects neurotransmitter activity and cerebral blood flow, causing headaches.^9^, ^10^ The reduction in sex hormones also leads to vasodilation, resulting in vasomotor symptoms such as hot flushes and hyperhidrosis.^11^ Additionally, a localised reaction at the injection site, presenting as pain, swelling and erythema, may also be encountered by some individuals.^11^

Hot flushes may be best treated with Serotonin Noradrenaline Reuptake Inhibitors (i.e. Venlafaxine) or Selective Serotonin Reuptake Inhibitors (i.e. Sertraline). Although hormonal treatments such as megestrol acetate or cyproterone acetate may be used in other patient population groups (i.e. prostate cancer patients taking androgen deprivation therapies), they are not employed in this population. Hyperhidrosis is temporary, but treatment options include strong topical antiperspirants (aluminium chloride 20%), oral anticholinergics or intradermal botulinum toxin injection. Injection site reactions are self‐limited but may be managed with a cool compress and analgesia.

### Stage 2 treatment (gender‐affirming treatment)

#### Xerosis and dermatitis (affirmed females)

Androgens are the primary driver of sebum production and acne formation.^12^ Oestradiol exerts a negative feedback loop on the gonadal axis, thereby decreasing androgen production.^12^ This is corroborated by studies demonstrating reduced sebum output in affirmed females (MTF) undergoing oestradiol and anti‐androgen therapy. These therapies may improve acne but potentially induce generalised xerosis, eczematous changes and pruritus.^13^, ^14^, ^15^ Fortunately, typical eczema management strategies, including regular emollients and topical corticosteroids, may be helpful in such cases.

#### Acne and Seborrhoea (affirmed males)

Testosterone therapy in affirmed males (FTM) can exacerbate acne, particularly on the face and back.^15^ The exact mechanisms remain unclear, but existing acne patients often have higher testosterone and 5α‐dihydrotestosterone (DHT) levels.^16^ It is hypothesised that exogenous testosterone increases local conversion to 5α‐DHT, which binds to androgen receptors and stimulates sebum production.^17^

Many FTM individuals experience an initial worsening of acne within the first 4 months, peaking around month six, often subsiding within a year.^18^ Chest‐binding practices may exacerbate acne due to friction. Topical therapies and systemic antibiotics may be insufficient, necessitating more aggressive therapies to prevent scarring.^19^ Spironolactone is contraindicated due to potential interference with testosterone therapy. Combined Oral Contraceptive Pills (COCPs) offer a safe contraceptive option for FTM individuals undergoing testosterone therapy. The low‐dose oestrogen does not significantly affect masculinisation.^20^ However, some transgender youth may prefer non‐oestrogen‐containing medications.

Isotretinoin remains the most effective treatment for patients undergoing masculinising therapy, as it specifically targets the androgen's impact at the level of the sebaceous gland.^17^ While isotretinoin can be beneficial, its use presents some drawbacks. For instance, isotretinoin has a potential risk of wound healing disruption and increases the likelihood of keloid formation in patients seeking top/bottom surgery.^21^ It is thought that the risk of hepatotoxicity from concomitant administration of isotretinoin and testosterone therapy has been overstated, and therefore liver monitoring should not differ from current guidelines for each medication.^20^ The increased psychiatric burden noted in TGD individuals may necessitate a more cautious approach and close communication with their treating psychiatrist, due to the anecdotal evidence of mental health disturbances with isotretinoin.^1^

For affirmed males with an intact uterus, pregnancy prevention counselling is crucial before and during isotretinoin use. While testosterone reduces fertility in affirmed males, it does not guarantee sterility. As previously mentioned, COCPs offer a safe contraceptive option for FTM individuals.

#### Patterned hair loss (developing in affirmed males and pre‐existing in affirmed females)

The term ‘patterned hair loss’ is preferred when discussing individuals with TGD identities, avoiding gender‐specific terminology such as ‘female pattern hair loss’. The pathogenesis of PHL involves the influence of androgens and exhibits a significant genetic component, which can manifest prior to gender affirmation in affirmed females.^22^ Conversely, affirmed males undergoing testosterone therapy face an increased susceptibility to PHL.^22^ Treatment algorithms need adaptation to ensure compatibility with interventions for gender dysphoria.

Certain interventions are applicable across gender identities. Minoxidil, a vasodilatory potassium channel opener, can prolong the anagen phase and facilitate hair follicle enlargement.^23^ Both topical minoxidil 5% and oral formulations are viable options for TGD individuals, though affirmed males may require higher doses.^23^ Low‐level light therapy (LLLT) enhances scalp blood flow, stimulates follicular stem cells and keratinocytes, and mitigates inflammation, synergising effectively with minoxidil.^24^, ^25^ Autologous Platelet Rich Plasma (PRP) deliver growth factors and bioactive proteins to the thinning scalp, demonstrating efficacy in both cisgender and transgender populations, particularly when used in conjunction with minoxidil.^23^, ^26^

The utilisation of certain hormonal therapies in TGD patients remains contentious.

#### Pharmacological interventions (finasteride, dutasteride and spironolactone)

Finasteride acts as a selective inhibitor of the 5a‐reductase type II isoenzyme, effectively halting the conversion of testosterone into DHT in the scalp and serum. This inhibition leads to the prolongation of the anagen phase, thereby stimulating hair growth.^23^ Neither the topical nor oral forms of finasteride lowers serum testosterone levels, making it particularly suitable for affirmed males seeking treatment without affecting their overall testosterone levels.^27^ However, some guidelines recommend a waiting period of 2–5 years to allow for the development of secondary sexual characteristics. Adverse effects of finasteride may include gynecomastia, temporary decrease in libido, erectile dysfunction and depression.^23^

In contrast to finasteride, dutasteride inhibits both isoenzymes of 5a‐reductase. Although it shares the same safety profile as finasteride, dutasteride may be more effective.^28^ As dutasteride is currently used off‐label to treat PHL, it should be reserved for affirmed males in whom finasteride has failed.

Spironolactone, an androgen receptor blocker, is sometimes used as a hormonal therapy in affirmed females. Due to its mechanism of reducing testosterone levels, it is generally avoided in affirmed males.

#### Surgical interventions (hairline advancement and transplant)

Hairline advancement is a surgical procedure performed on affirmed females aiming to achieve a more feminine appearance. This procedure typically involves the inferior migration of the natural hairline by an average of 2 cm.^23^

In cases where other treatment options have been ineffective, hair transplantation stands out as an exceptional long‐term solution for both affirmed males and females with severe PHL. To maximise outcomes and minimise postoperative PHL progression, it is recommended that patients initiate oral finasteride and/or topical application of minoxidil 4 weeks prior to the transplantation and continue this regimen for 48 weeks postoperatively.^29^

#### Hypertrichosis/hirsutism (developing in affirmed males and pre‐existing in affirmed females)

**FIGURE 2:**
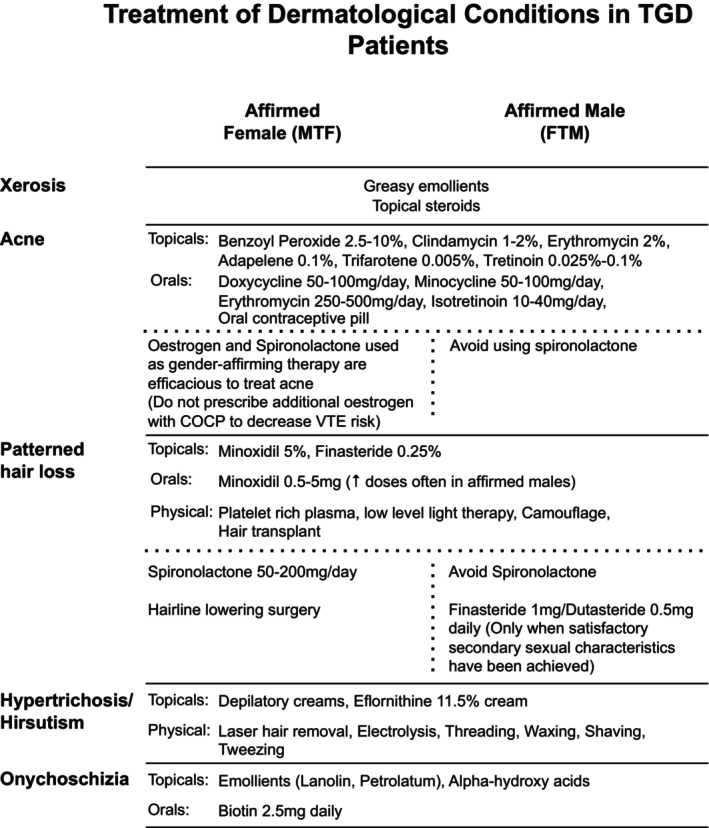
Treatment of dermatological conditions in TGD Patients.

Affirmed females who take oestrogen and antiandrogen therapy can experience a reduction in both terminal hair growth rate and density, with more pronounced effects on body hair than facial hair.^15^ Although Ferriman and Gallwey scores typically decrease during feminising therapy, most patients remain above a score of seven, which categorises them as hirsute.^30^ The hair diameter, growth rate or density for affirmed females taking feminising therapy alone, will not fall to the same values as cis‐gendered females.^30^ Dermatologists may be of assistance in aiding with the treatment of perceived hirsutism (Figure 2).

In contrast, testosterone has the opposite effect of increasing body and facial hair growth.^30^ This increase in hair growth is noticeable within the first 6 months of starting testosterone with most reaching a Ferriman and Gallwey score of above eight, just below the average cis‐gendered males score of 10.5.^30^ Unfortunately, use of testosterone may also result in PHL.

For individuals seeking permanent hair removal solutions, electrolysis is particularly effective for blonde hair, whereas laser hair removal is most suitable for those with light skin and dark hair.^31^ For temporary measures, dermaplaning removes fine hair using a sterile scalpel, while depilatory creams utilise chemicals to break down hair protein, offering convenience and ease of use. Prior to 2018, Eflornithine Hydrochloride was commercially available in Australia to slow hair growth but is now only accessible through compounding. Temporary physical methods, such as waxing, threading and shaving, are other options.

#### Onychoschizia

At the time of writing, a few papers have noted a causation of oestrogen use resulting in brittle nails.^14^, ^32^ It is theorised that anti‐androgenic effects of gender‐affirming therapy can lead to a decreased activity of sebaceous glands resulting in brittle nails.^32^

#### Connective tissue disease

The development of Systemic Lupus Erythematosus (SLE) in affirmed females following the short‐ and long‐term oestradiol has been reported.^33^ Furthermore, the case of an affirmed male with SLE showing improvement in symptoms after initiation of testosterone therapy has been published.^34^ This suggests a correlation between the use of exogenous oestrogen and the manifestation of SLE.

### Stage 3 treatment (gender‐affirming surgery/procedures)

#### Gender reassignment surgeries

Upon reaching an appropriate age, typically around 18 years, TGD individuals may elect to pursue gender reassignment surgeries. These irreversible surgeries are generally reserved for TGD individuals who have consistently lived in their identified gender role for a minimum of 12 months.^3^

Vaginoplasty utilises penile or scrotal skin for neovaginal creation.^19^ However, this approach can lead to intravaginal hair growth, which can be a nidus for infection.^35^, ^36^ While permanent hair removal is imperfect, electrolysis and laser hair removal are preferred pre‐surgical options.^36^ Therefore, it is recommended that patients wait at least 3 months after the last hair removal procedure before undergoing vaginoplasty to minimise the risk of intravaginal hair growth.^36^

The skin used to create neovaginas is vulnerable to human papillomavirus (HPV) infection, which can lead to the development of condyloma acuminata and squamous cell carcinoma.^37^ HPV types 16, 31 and 33 have been identified as the most common variants associated with these conditions.^37^ Therefore, it is essential to perform regular examinations of the internal and external neogenitalia to monitor for any signs of disease and to ensure that individuals are vaccinated against HPV prior to undergoing surgery.

Published articles report the manifestation of genital lichen sclerosus in the neovagina, with one study noting its development 8 years after gender reassignment surgery in a transwoman taking oestradiol and finasteride.^38^ This highlights the importance of close monitoring for skin cancer surveillance in the neovagina. Additionally, rare cases of mucinous adenocarcinoma have been reported in a neovaginas constructed from colon tissue.^39^

Neophallus creation via metoidioplasty or phalloplasty utilises donor skin from the labia majora/minora, thigh, abdomen or forearm.^19^ This can increase the risk of skin cancer, particularly in areas with significant sun exposure, necessitating close surveillance. Urethral complications, such as strictures and fistulas, can arise and impact surrounding tissues leading to dermatitis.^40^ Additionally, hypertrophic scars may develop at the graft sites.

#### Cosmetic injectables

**FIGURE 3:**
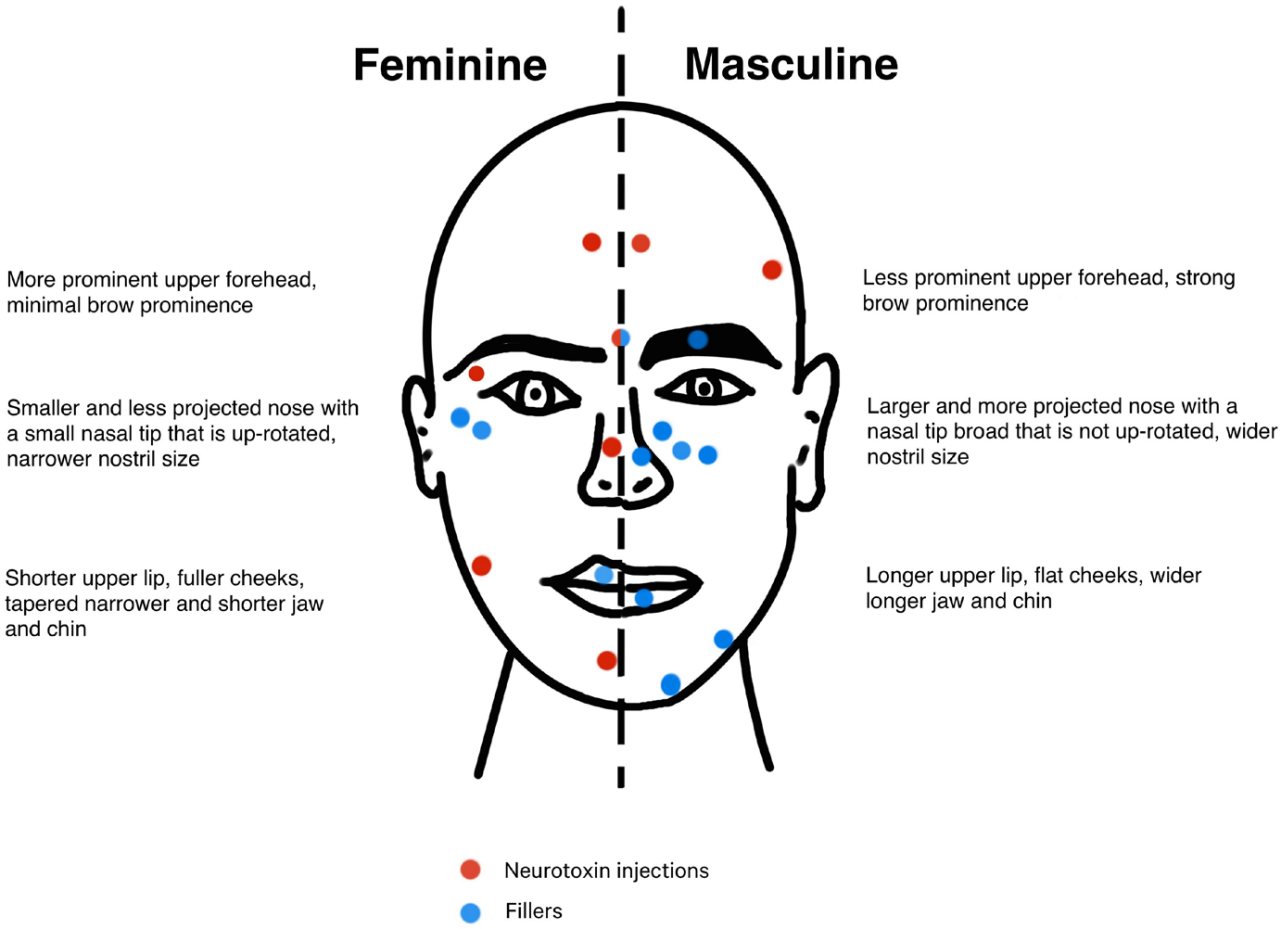
Comparison of feminine and masculine features with sites for neurotoxin injection and fillers. Adapted from Dhingra.^42^

In Australia, regulations classify cosmetic injections as Schedule 4 substances, requiring assessment and prescription by a medical professional. Dermatologists can provide gender‐affirming injectable treatments by the use of TGA approved fillers and botulinum toxin injections to shape the face accordingly to the patient's demands (Figure 3).^41^

Facial feminisation or masculinisation through cosmetic modifications is a common desire among TGD individuals to achieve facial features associated with their affirmed gender.

Currently, no research explores the illegal use of cosmetic injectables within the Australian TGD population. Overseas, the illegal use of cosmetic injectables varies widely among TGD communities, with rates ranging from 16% in the United States of America to as high as 68.6% in Thailand.^43^ These procedures often utilise inexpensive and potentially harmful materials like silicone, oil and paraffin.^42^ Furthermore, the non‐sterile conditions in which the procedures are frequently performed, increase the risk of life‐threatening consequences, such as foreign body reactions, chronic infections due to biofilm formation and foreign body pulmonary emboli.^42^, ^44^, ^45^, ^46^ Silicone injectables in particular have been shown to cause silicone granulomas, which typically require surgical intervention as the first line of treatment. However, given the migratory nature of silicone injectables, medical interventions such as systemic steroids, hydroxychloroquine and tetracycline have proven to be more efficacious, with surgical interventions reserved as a second line option.^47^

#### Body contouring

Body contouring procedures can help TGD individuals achieve a physical appearance that aligns with their gender identity. Affirmed females may opt for breast augmentation with implants to create a more feminine silhouette. Conversely, affirmed males can undergo mastectomy, which removes breast tissues for a more masculine chest contour.

To achieve a desired waist–hip ratio, TGD individuals have options like targeted fat removal via liposuction and autologous fat grafting, which utilises the patient's own fat for redistribution.^48^ For non‐invasive approaches, cryolipolysis, radiofrequency and ultrasound devices are available.

## CONCLUSION

This area of medicine is rapidly changing, particularly in Australia where a large medical insurance company last year announced it would no longer provide indemnity for GPs initiating gender‐affirming care.^49^ The provision of gender‐affirming care draws many differing opinions and can sometimes even prove challenging for clinicians working in this space.^50^ TGD individuals may have greater obstacles than many patients in accessing dermatological care due to the financial implications of multiple medical appointments, often worse psychological health and previous negative interactions with the medical system.^6^ Continuous research and advocacy activities are critical to raise awareness and promote inclusivity in dermatological care for the TGD population in Australia.

## CONFLICT OF INTEREST STATEMENT

We have no known conflict of interest to declare.

## Data Availability

Data sharing not applicable to this article as no datasets were generated or analysed during the current study.
